# The Role of Mitochondria in Cardiovascular Diseases

**DOI:** 10.3390/biology9060137

**Published:** 2020-06-25

**Authors:** Anastasia V. Poznyak, Ekaterina A. Ivanova, Igor A. Sobenin, Shaw-Fang Yet, Alexander N. Orekhov

**Affiliations:** 1 Department of Basic Research, Institute for Atherosclerosis Research, Skolkovo Innovative Center, 121609 Moscow, Russia; tehhy_85@mail.ru (A.V.P.); kate.ivanov@gmail.com (E.A.I.); 2 Laboratory of Medical Genetics, National Medical Research Center of Cardiology, 15A 3-rd Cherepkovskaya Street, 121552 Moscow, Russia; igor.sobenin@gmail.com; 3 Laboratory of Cellular and Molecular Pathology of Cardiovascular System & Central Laboratory of Pathology, Institute of Human Morphology, 3 Tsyurupa Street, 117418 Moscow, Russia; 4 Institute of Cellular and System Medicine, National Health Research Institutes, 35 Keyan Road, Zhunan Town, Miaoli County 35053, Taiwan; syet@nhri.org.tw; 5 Laboratory of Infection Pathology and Molecular Microecology, Institute of Human Morphology, 3 Tsyurupa Street, 117418 Moscow, Russia; 6 Laboratory of Angiopathology, Institute of General Pathology and Pathophysiology, 8 Baltiyskaya st., 125315 Moscow, Russia

**Keywords:** atherosclerosis, mitochondria, mitochondrial genome, cardiovascular disease

## Abstract

The role of mitochondria in cardiovascular diseases is receiving ever growing attention. As a central player in the regulation of cellular metabolism and a powerful controller of cellular fate, mitochondria appear to comprise an interesting potential therapeutic target. With the development of DNA sequencing methods, mutations in mitochondrial DNA (mtDNA) became a subject of intensive study, since many directly lead to mitochondrial dysfunction, oxidative stress, deficient energy production and, as a result, cell dysfunction and death. Many mtDNA mutations were found to be associated with chronic human diseases, including cardiovascular disorders. In particular, 17 mtDNA mutations were reported to be associated with ischemic heart disease in humans. In this review, we discuss the involvement of mitochondrial dysfunction in the pathogenesis of atherosclerosis and describe the mtDNA mutations identified so far that are associated with atherosclerosis and its risk factors.

## 1. Introduction

Accumulating evidence places mitochondrial dysfunction as a factor playing a central role in the pathogenesis of several chronic human diseases, including cardiovascular disease (CVD) [1]. Under normal conditions, mitochondria serve as cellular powerhouses responsible for ATP production, with reactive oxygen species (ROS) being generated as byproducts that are rapidly neutralized by cellular antioxidant systems. Dysfunctional mitochondria not only compromise cellular respiration and energy production, but also act as dangerous ROS generators leading to oxidative stress, and as triggers of apoptosis or cell damage and death. These processes were described as important components of the pathogenesis of atherosclerosis and associated CVD [2]. Vascular wall cells are especially sensitive to oxidative stress and are designed to respond to rapid changes in the environment, such as altered oxygen levels and the presence of pathogens and endogenous damaging stimuli. Moreover, vascular endothelial cells (ECs) are in close contact with circulating immune cells that initiate immune reactions, thereby affecting the vascular wall. Endothelial dysfunction and local activation of the ECs leading to recruitment of immune cells appears to be one of the earliest stages of atherosclerosis development, preceding atherosclerotic plaque formation. Mitochondria participate in all these processes, leading to their identification as an attractive possible point of therapeutic intervention [3].

Atherosclerosis of vital arteries, such as the aorta and the coronary arteries, is the underlying cause of the most prevalent and dangerous CVD. Despite the continuing search for novel medicines and improving healthcare, the risk of developing CVD is consistently high in developed countries. For instance, more than 4 million Europeans die every year, of which 55% are women and 45% are men, although a higher mortality rate from CVD under the age of 65 is associated with male sex. The economic costs associated with temporary and permanent disability among the working population in Europe amounted to about 106 billion euros in 2009. Therefore, atherosclerosis remains an important socioeconomic problem. Accumulating evidence shows that the disease can develop asymptomatically for a long time, affecting much younger populations than previously thought [4]. Given the deleterious effects and the high costs of treatment of atherosclerotic consequences, focusing on disease prevention and the improvement of diagnostic methods for early detection should be the priority of the future studies [5].

Several recent studies demonstrated the association between overall damage and the presence various mitochondrial DNA (mtDNA) mutations with cardiovascular disease [6,7,8]. Atherosclerosis can be characterized as multifactorial disorder associated with hyperlipidemia and chronic inflammation [9,10]. Nonmodifiable risk factors of atherosclerosis include older age, male sex, environmental influences, and genetic factors contributing to dyslipidemia, hypertension, glucose tolerance, diabetes mellitus, and obesity. Anatomical, physiological, and metabolic (biochemical) features of atherosclerosis include dyslipidemia, arterial hypertension, obesity, and diabetes. Behavioral (modifiable) risk factors of atherosclerosis include inappropriate dietary habits, smoking, reduced physical activity, alcohol consumption, and stress. It is difficult to assess the relative contribution of each of these factors to atherosclerosis development or define their relative impact depending on the patient’s age. Numerous genetic variants are associated with the pathology, with genetic susceptibility appearing to vary across human populations. It is likely that atherosclerosis development depends more on gene–gene and gene–environment interactions rather than on individual genes or factors [11].

The disease can affect any artery, causing profound changes and loss of functionality of the arterial wall, described as an atherosclerotic lesion. According to current understanding, the process begins with local endothelial dysfunction, characterized by increased permeability and activation of the endothelium, which starts to express adhesion molecules that facilitate the recruitment of circulating immune cells. The local inflammatory response plays a central role in atherosclerosis pathogenesis [12]. The emerging atherosclerotic lesion becomes accessible to circulating lipoprotein particles that penetrate the arterial wall and become internalized by cells, causing local lipid accumulation. An altered blood lipid profile is one of the earliest recognizable risk factors of atherosclerosis, as it is the low-density lipoprotein (LDL) fraction that serves as the main source of lipids accumulating in the arterial wall [13]. Mitochondria play a prominent role in overall lipid metabolism in the body, being the site of β-oxidation of free fatty acids [14]. Furthermore, they are involved in every step of atherogenesis, from local endothelial dysfunction to the formation of unstable plaques [15].

The deleterious effects of mitochondrial dysfunction are not limited to atherosclerotic plaque development in the vascular wall but can be expanded to other parts of the cardiovascular system. Cardiomyocytes characterized by high energy consumption that are tightly dependent on correct mitochondrial functioning appear to be especially sensitive to aberrations of the mitochondrial turnover process [16]. Deficient biogenesis of mitochondria was also shown to be associated with endoplasmic reticulum (ER) stress involved in the pathogenesis of abdominal aortic aneurysm [17]. In this review, we discuss the emerging role of mitochondria in the pathogenesis of cardiovascular diseases, the significance of mtDNA mutations as potential diagnostic markers, and the potential use of mitochondria as therapeutic targets.

## 2. Mechanisms of Mitochondrial Dysfunction

Accumulating evidence is revealing novel links between mitochondrial dysfunction and CVD pathogenesis. In order to better understand the mechanisms of this interplay, it is important to outline the main pathways of mitochondrial damage, which involve genetic factors (mtDNA damage), incorrect functioning of the respiratory chain, and impairment of mitochondrial turnover. Mitochondria possess circular genomes resembling bacterial chromosomes [18]. The double-stranded, circular mtDNA is around 16.5 thousand nucleotides long and is maintained by mitochondrial enzymes that perform synthesis and repair. The mitochondrial genome encodes 37 genes, including those that are necessary for energy production in the mitochondrial respiratory chain, including 13 structural genes encoding subunits of oxidative phosphorylation complexes, 22 transport RNAs (tRNAs), and 2 ribosomal RNAs (rRNAs) [19]. Like bacterial chromosomes, mtDNA exist in several copies inside one organelle. The mutations occurring in mtDNA can therefore be homoplasmic, where all mtDNA copies are identical, or heteroplasmic, where only some mtDNA copies carry a certain mutation [20]. Mitochondrial mutations can accumulate during the life of an individual, forming a carrier phenotype. Many mtDNA mutations were shown to be associated with human disorders, including CVD, which is discussed in detail below. The impact and distribution of mitochondrial mutations vary widely and depend on several factors, with heteroplasmy playing a major role [21].

Mitochondria are potent regulators of apoptosis, since mitochondrial damage and loss of mitochondrial membrane integrity leads to the release of apoptotic factors and caspase activation [22]. Moreover, mitochondria participate in maintaining Ca^2+^ homeostasis. Transfer of Ca^2+^ from the ER, which serves as primary cellular Ca^2+^ reservoir, can lead to mitochondrial dysfunction and fragmentation of the organelle [23]. Functioning of the mitochondria and the ER are tightly linked in the cell. Perturbations of mitochondrial lipid metabolism can activate the unfolded protein response (UPR) and ER stress [24]. Notably, CVD risk factors such as hyperlipidemia and exposure to modified LDL (oxLDL) are among the known triggers of mitochondrial dysfunction [25].

ATP synthesis in the mitochondria includes the following stages: Conversion of pyruvate and fatty acids received from the cytoplasm into acetyl-CoA, oxidation of acetyl-CoA in the Krebs cycle, leading to the formation of nicotine amide adenine dinucleotide (NADH), electron transfer from NADH to oxygen through the respiratory chain, and ATP synthesis by the membrane ATP synthetase complex [26]. Oxidative phosphorylation accompanying ATP production also acts as an endogenous source of ROS. Regulatory effects of ROS in the cell include inactivation of the Fe-S centers of the enzymatic complexes responsible for oxidative phosphorylation and the tricarboxylic acid cycle enzyme aconitase, leading to a reduction in ATP production. However, long-term exposure to ROS or excessive ROS generation can lead to oxidative damage to proteins, lipids, and nucleic acids [27]. Oxidative stress associated with excessive ROS formation is an important component of atherosclerosis pathogenesis, as explained further [2]. The effects of ROS are currently recognized as major drivers of mtDNA mutagenesis, which leads to accumulation of multiple mutations, a decrease in the rate of oxidative phosphorylation, and an even greater accumulation of ROS, thereby forming a vicious circle. However, ROS-induced damage is not the only driver of mtDNA mutagenesis. Replication errors introduced by mitochondrial DNA polymerase γ (POLG) make up another major source of mutations [28]. Moreover, the mitochondrial DNA repair system is far less efficient than the nuclear system. Unlike nuclear DNA, mtDNA is not protected by histone packaging, thereby further contributing to mutagenesis resulting from DNA damage. Instead, mtDNA is associated with a number of proteins that help maintain its function and offer some level of protection [29]. Taken together, the abovementioned features of mtDNA contribute to the much higher mutation rate observed in comparison to nuclear DNA [30]. Thus, the balance of mitochondrial oxidizing agents is a key regulator of cell life and controller of mtDNA mutation accumulation [31].

The process of mitochondrial biogenesis and turnover ensures the correct distribution of mitochondria between dividing cells and maintenance of the mitochondrial population within the cell, corresponding to current energy demand. Moreover, mitochondrial turnover allows for the removal of dysfunctional and damaged organelles and replacement with functional ones. In brief, mitochondria undergo cycles of fission and fusion, during which dysfunctional parts of the organelles can be cut off into small spherical fragments that are subsequently degraded by mitophagy. Unaffected parts of the mitochondria fuse with each other, forming elongated, functional organelles [32]. The main proteins responsible for mitochondrial fission are dynamin-related protein 1 (Drp-1) and mitochondrial fission protein 1 (Fis-1). Mitochondrial fusion is mediated by mitofusins 1 and 2 (Mfn-1 and 2) and optic atrophy 1 (Opa-1). The activity of these proteins is regulated by several pathways, such as ubiquitination and sumoylation. Imbalance between the activities of these groups of proteins leads to deficient or excessive fragmentation of the mitochondria and impaired mitophagy, and was shown to be involved in the development of different human pathologies [33]. Disruption of mitochondrial turnover promotes cell damage and apoptosis. Disturbances of mitochondrial turnover were demonstrated in several CVD, including atherosclerosis, reperfusion injury, cardiomyopathy, and cardiac hypertrophy [16]. The processes of mitochondrial turnover and apoptotic cell death could be explored for the development of potential new therapies [34]. It is important to understand why mtDNA mutations, both heteroplasmic and homoplasmic, persist in cells and tissues despite the functioning processes of mitochondrial turnover. At least three hypothetical explanations are suggested. First, the fusion/fission system becomes overloaded and loses functionality. Second, mitophagy is inhibited in cells suffering from mitochondrial dysfunction, even though fission/fusion processes are intact. Finally, in affected cells, the process of mtDNA replication followed by division of organelles is launched as a primary response to ATP pool depletion. In this case, both mutant and normal mtDNA molecules would be replicated, thus sustaining the proportion of heteroplasmic and homoplasmic variants, although it is not known whether their replication rates are similar. Experimental testing of the above hypotheses is needed to bring clarity to this question. Although it is still too early to evaluate the possible clinical use of mitochondrial fission and fusion regulation, some encouraging results are starting to appear; for instance, a recent study in a mouse model employed the pharmacological mitochondria fission inhibitor mdivi1, as well as genetic inhibition of fission through down-regulation of Drp-1, to alleviate abdominal aortic aneurysm [35].

To date, several severe human pathologies have been identified as being dependent on mitochondrial dysfunction caused by certain mtDNA, as well as nuclear DNA mutations. These so-called mitochondrial diseases constitute a large, heterogeneous group of hereditary diseases associated with alterations in mitochondrial structure and functioning and deficiency of respiratory processes in tissues [1]. The range of clinical manifestations of mitochondrial diseases varies from moderate subclinical to severe symptoms that can reduce life expectancy. Moreover, mitochondrial diseases are among the most common neurogenic illnesses. The most studied mitochondrial diseases caused by mutations in both nuclear DNA and mtDNA include Mitochondrial Encephalomyopathy, Lactic Acidosis, and Stroke-like episodes (MELAS syndrome), Myoclonic epilepsy with ragged-red fibers or Cushing’s syndrome (MERRF syndrome), Kearns–Seir Syndrome (KSS), Bart syndrome, Leber’s Hereditary Optic Neuropathy (LHON), Chronic Progressive External Ophthalmoplegia (CPEO), Mitochondrial Myopathy (MM), and Pearson syndrome, among others. The study of mitochondrial diseases is a rapidly developing research area, but effective treatments for them still remain to be found. One of the challenges in the development of such therapies is the necessity for targeted delivery of active agents to affected mitochondria [36].

## 3. Mitochondrial Dysfunction and Oxidative Stress in Atherogenic Processes

Mitochondrial dysfunction is believed to be associated with numerous cardiac diseases, such as atherosclerosis, ischemia–reperfusion injury, heart failure, and hypertension, presumably due to insufficient cellular energy production and uncontrolled production of ROS. Being a crucial phenotypic characteristic of mitochondrial dysfunction, mtDNA damage also plays an important role. Mitochondrial dysfunction is characterized, among other features, by the absence or dysfunction of mitochondrial enzymes, impaired mitochondrial biogenesis, decreased numbers of mitochondria in tissues, stimulation of apoptosis and inflammation, stimulation of fibrosis and cardiac remodeling, and impairment of sarcomere protein function [37,38]. All of these impairments are potential mechanistic factors for ischemic heart disease and heart failure development, both in terms of pathogenesis and progression. However, their implications in disease pathogenesis need to be further confirmed by clinical and experimental data.

Oxidative stress, which is closely associated with mitochondrial dysfunction, is recognized as one of the factors of atherosclerosis initiation and progression. The signaling role of ROS is prominent in vascular system cells, such as ECs, vascular smooth muscular cells (VSMCs), and fibroblasts [39]. At low concentrations, ROS are involved in vascular signaling processes and regulate the activity of mediating proteins, enzymes, and ion channels in ECs. Exposure to stress conditions, such as shear stress or the presence of vasoactive factors, results in increased ROS generation in ECs and VSMCs. Importantly, known atherosclerosis risk factors, such as hyperlipidemia, smoking, and hypertension, are also potent inducers of ROS in vascular tissues (Table 1) [8,40,41].

It was demonstrated that elevated LDL and the presence of atherogenic forms of LDL, for instance, due to oxidation modification, can cause mitochondrial damage [25]. Macrophage overload with free cholesterol was associated with pronounced mitochondrial dysfunction and triggering of apoptosis [42]. Decreases transmembrane potential of mitochondria and activation of the mitochondrial apoptosis pathway could be assumed to contribute to this effect. OxLDL induces increased mitochondrial ROS production in ECs associated with apoptosis by activating mitochondrial complex II and NADH oxidase [43]. Moreover, besides ECs, this process was described in all cell types involved in atherosclerosis, i.e., VSMCs, macrophages, and T-lymphocytes [44].

Hypertension, another well-known risk factor of atherosclerosis, is associated with endothelial dysfunction and oxidative stress [46]. Moreover, hypertension is associated with the malfunction of mitochondrial ATP synthase in cardiomyocytes. In addition, mitochondrial Ca^2+^ overload significantly contributes to the development of hypertension [47].

Cigarette smoking significantly increases the risk of early atherosclerosis. In addition to endothelial damage, platelet activation, and LDL cholesterol oxidation, the atherogenic effects of smoking are also mediated by oxidative damage to mtDNA with the appearance of mtDNA deletions and loss of mitochondrial membrane potential [45].

In the endothelium, stress factors can lead to uncoupling of endothelial NO synthase (eNOS), which is the main producer of nitric oxide (NO), leading to generation of the superoxide anion in place of NO and resulting in increased ROS production in ECs and VSMCs. Experimental studies showed that oxidative damage caused by mitochondria is the mechanism underlying the development of age-associated endothelial dysfunction. Mitochondria-targeting antioxidants, such as MitoQ, appear to be promising for the preservation of vascular endothelial function with age and the prevention of CVD development [48]. Studies in mice demonstrated that deficiency of superoxide dismutase results in mtDNA damage and accelerates the development of atherosclerosis. High levels of ROS were detected in macrophages and smooth muscle cells in the atherosclerotic plaques of model animals, thereby providing evidence that oxidative DNA damage and repair increase significantly in atherosclerotic plaques [49].

Excessive ROS production is recognized as a key mechanism by which mitochondria are involved in the development of CVD, such as coronary heart disease, cardiomyopathy, ischemia–reperfusion-associated damage, heart failure, and arrhythmia. ROS can react with cell components such as polyunsaturated fatty acids, proteins, nucleic acids, and carbohydrates, violating the normal properties of cell membranes (such as efficient ion transport), changing enzyme activity, and interfering with protein synthesis and transport. Together, these alterations can lead to profound cellular stress and apoptosis. Under conditions of mitochondrial dysfunction, excessive production of reactive oxygen and nitrogen species contributes to inflammatory vascular reactions, leading to the development of atherosclerotic lesions [50]. Active forms of oxygen and nitrogen play important roles in atherogenesis by promoting EC dysfunction and apoptosis, activating matrix metalloproteinases, VSMC growth and intima migration, expressing adhesion molecules, and LDL oxidation [35]. During advanced stages of the disease, apoptosis of VSMCs, induced in part by oxidative stress, is potentially involved in plaque instability and rupture [51]. Other known risk factors of coronary heart disease were shown to be associated with increased ROS production [52].

Hyperglycemia is an important factor in cardiovascular system damage, previously shown to promote mitochondrial dysfunction and ER stress. ROS generated as a result of these changes can directly damage lipids, proteins, or DNA and modulate intracellular signaling pathways, such as mitogen-activated protein kinases and redox-sensitive transcription factors, causing changes in protein expression and, therefore, irreversible oxidative modifications. Such conditions can form in case of poorly managed diabetes mellitus, which is a well-known risk factor of CVD [39].

Evidence accumulated so far positions mitochondrial dysfunction centrally regarding the pathogenesis of CVD. It is possible that mitochondria provide a unifying link explaining the atherogenic effect of different CVD risk factors. Targeting mitochondrial dysfunction for treatment of CVD became a rapidly developing research direction over recent years, with both alleviation of mitochondrial oxidative stress and the influence of signaling pathways currently being explored [48]. For instance, a protective role of dissociating protein 2 (UCP2) against mitochondrial ROS-induced endothelial dysfunction was revealed. Activation of transient receptor potential of vanilloid 1 (TRPV1), an ion channel that responds to elevated temperature involved in pain sensation, alleviated vascular dysfunction through the UCP2 protein kinase pathway. Administration of capsaicin, a ligand that activates TRPV1, to model mice kept on a high-fat diet improved animal survival and had a positive effect on coronary function, which was dependent on intact expression of both the TRPV1 and UCP2 genes [53].

## 4. Mitochondrial DNA Mutations Associated with Cardiovascular Pathologies

Mitochondrial mutations can occur both in somatic and germline cells [54]. Mutations in somatic cells lead to a decrease in energy production, and mutations that occur in germline cells can be transmitted to following generations and lead to the development of several hereditary human diseases, including atherosclerosis. MtDNA is inherited through the maternal line. Women that carry deleterious mtDNA mutations can therefore transfer them to their children. Somatic mutations occur in the mitochondrial genome throughout the life of an individual and are transferred along a cell line in the body [55]. The effects of different mtDNA mutations may vary significantly depending on the nature of the affected gene and the level of heteroplasmy. Certain mtDNA mutations manifest themselves only when the level of heteroplasmy reaches a certain value, which is followed by a disruption of mitochondrial function and cellular energy deficiency and damage. Cells and tissues with naturally high energy consumption, such as cardiomyocytes, appear to be more sensitive to deleterious mtDNA mutations. The heteroplasmy level of mtDNA mutations determines the severity of mitochondrial disease such as atherosclerosis, which is known as the “threshold effect” [56].

Accumulation of mtDNA mutations may be reflected by alterations in mitochondrial structure and function that can be observed microscopically. The presence of such altered mitochondria is demonstrated in atherosclerotic lesions. Moreover, the distribution of ECs with dysfunctional mitochondria in blood vessel endothelia may at least partially explain the focal appearance of atherosclerotic lesions. This hypothesis was tested in a study that compared intact aortic intima areas and lipofibrous plaques obtained from 12 postmortem aorta samples to identify the average level of several heteroplasmic mtDNA mutations, including m.1555A>G, m.3256C>T, m.12315G>A, m.14459G>A, and m.15059G>A. It was shown that four mutations in the mitochondrial genome (m.1555A>G within the MT-RNR1 gene, m.3256C>T in the MT-TL1 gene, m.12315G>A in the MT-TL2 gene, and m.15059G>A in the MT-CYB gene) were more prevalent in lipofibrous plaques compared to unaffected aortic intima, thereby confirming the likely role of these mutations in atherosclerosis development [57].

Another study employed parallel sequencing of mtDNA from myocardial tissues of patients with coronary artery disease and compared them to samples obtained from control individuals. The results showed that in patients with coronary artery disease, the level of mtDNA heteroplasmy was 39.8% higher than in control myocardial tissues. The study also found that the total number of heteroplasmic mtDNA deletions in patients with coronary artery disease was 87% higher compared to the control. A similar trend was observed regarding single nucleotide variants in mtDNA. Tissues from patients with ischemic heart disease were found to contain increased levels of heteroplasmic mtDNA variants at 41.4% higher than control levels, with increased mtDNA heteroplasmic deletions, as high as 87.50%, and increased in heteroplasmic variants of single nucleotide mtDNA, as high as 48.76% [58]. These observations indicate the increased somatic mutagenesis and accumulation of mtDNA mutations in atherosclerotic plaques compared to unaffected tissues.

A study conducted in 2010 explored the relationship of single nucleotide polymorphisms (SNPs) with the lysyl oxidase-like 1 (LOXL1) gene with pseudoexfoliation glaucoma (PEG) using LOXL1 coding region sequencing techniques [59]. The authors evaluated the role of product variants of m.16169T>C and various mtDNA haplogroups as risk factors for the development of ischemic heart disease. The study included 669 individuals from Saudi Arabia with angiographically confirmed atherosclerosis and 258 control individuals. In this population, carriage of the m.16189C minor allele was associated with an increased risk of atherosclerosis (1.524 (1.076–2.159), *p* = 0.017), although this association was influenced by other factors, such as age, history of myocardial infarction, and hypertension. Among haplogroups, only N1c showed a protective connection with coronary heart disease as an independent factor. This association was significant in the total sample (0.176 (0.042–0.736), *p* = 0.017) and in the age group <50 years (0.075 (0.008–0.743), *p* = 0.027), demonstrating that mtDNA polymorphisms contribute to the risk of atherosclerosis, depending on factors such as history of myocardial infarction, hypertension, and age.

The role of a large deletion in mtDNA affecting 4977 base pairs (ΔmtDNA (4977)) was explored by several studies. The deletion was detected in different cells, where it led to mitochondrial dysfunction. The ΔmtDNA (4977) affects several genes, including ND5, ND4, ND4L, ND3, and COXIII, as well as ATPase genes 6 and 8 and tRNA genes. As a result, seven polypeptide components of the respiratory chain and 5 of the 22 tRNAs are affected [60]. In patients with mitochondrial diseases, the overall copy number of ΔmtDNA (4977) and the proportion of mitochondrial genome bearing the deletion in circulating leukocytes was found to increase with age and correlated with symptom severity [60]. Recent studies of mutagenesis and mtDNA damage in atherosclerotic lesions provided evidence of somatic mtDNA mutation involvement in atherogenesis [61].

Several studies showed a link between mitochondrial SNPs (mtSNPs) and conditions such as myocardial infarction, atherothrombotic brain infarction, and diabetes. The contribution of mtSNPs to the development of acute CVD and atherosclerosis was demonstrated in a study conducted in Japan on human autopsy material [62]. The study included 1536 autopsy cases (827 men and 709 women) with an average age of 80 years. The authors investigated 149 mtSNPs using the PCR–Lumine method and accounted for age-related and normal cardiovascular risk factors using the logistic regression method, resulting in the identification of 36 haplogroups of interest. Among the 10 haplogroups with frequencies of >0.04, haplogroups A and M7a were found to be significantly associated with coronary atherosclerosis, with odds ratios (95% confidence intervals) of 1.80 (1.09–2.97; *p* = 0.023) and 1.92 (1.23–3.01; *p* = 0.004), respectively. Haplogroup D4a, which was previously reported to be associated with an extremely long lifespan in the Japanese population, was associated with pathological myocardial infarction in men, with an odds ratio of 2.05 (1.01–4.14; *p* = 0.046). This study demonstrated that mitochondrial haplogroups A and M7a are associated with a significant risk of developing coronary atherosclerosis in the Japanese population.

Another study aimed to establish the relationship between the development of early myocardial infarction (at 55 years of age) with angiographically shown coronary atherosclerosis [44]. The authors concluded that haplogroup H was associated with the early onset of myocardial infarction, but only among male smokers. This work confirmed the contribution of mtDNA polymorphisms to risk of atherosclerosis and ischemic events. The authors concluded that abnormalities of apoptosis contribute to lipid core formation in atherogenesis and depletion of VSMCs.

Population-specific mtDNA polymorphisms, especially when associated with changes in amino acid sequences of proteins, RNA structure, or regulatory sites in mitochondrial genomes, were also associated with CVD. For example, a study in an Iranian population showed that the m.750A>G polymorphism was 1.6 times more frequent in patients with coronary heart disease and was associated with increased risk of coronary heart disease development (OS = 1.6, 95% CI (0.24–3.01), *p* = 0.02) [63].

Several studies evaluated the possible relationships between mtDNA mutations and ion channel function deficiency. Such a link would connect mitochondrial dysfunction with arterial hypertension, one of the major risk factors of atherosclerosis. For instance, the mitochondrial m.4263A>G mutation in the tRNA-Ile gene was identified in a family with maternally inherited hypertension. This mutation could contribute to the impairment of mitochondrial Ca^2+^ homeostasis and transport through voltage-dependent anion channels [64]. Further investigations regarding the underlying mechanisms showed that the m.4263A>G mutation was associated with deficient mitochondrial Ca^2+^ uptake and cytoplasmic Ca^2+^ overload [65]. Moreover, several heteroplasmic and homoplasmic mutations were found in the mitochondrial COXII, ATP8, ATP6, ND1, tRNA-Lys, and tRNA-Gln genes, showing a statistical association with the risk of maternally inherited essential hypertension. Among the identified mtDNA mutations, the highest frequencies were detected for m.3970C>T, m.4048G>A, m.4071C>T, m.4086C>T, m.4164A>G, m.4248T>C, m.4386T>C, m.4394C>T, m.8414C>T, m.8701A>G, and m.8584G>A mtDNA variants and ΔmtDNA (8273_8281) deletion. The most affected genes appeared to be ND1 and ATP6 [66,67]. However, the precise mechanisms of the deleterious effect of mtDNA mutations on mitochondrial ion channels and cellular Ca^2+^ homeostasis remain unclear.

As many as 17 mtDNA mutations were found to be spread over genes encoding six tRNA, 12S rRNA subunits, and genes II and V of the NADH dehydrogenase subunits, which were reported to be associated with ischemic heart disease (Table 2) [68,69,70,71,72]. MtDNA mutations found in diseases such as nonfamilial forms of dilated and hypertrophic cardiomyopathy, MELAS syndrome, mitochondrial myopathy, and maternally inherited diabetes and deafness (MIDD) syndrome were also found to occur in coronary artery disease [73].

A study conducted in Russia on human autopsy material measured the heteroplasmy level of the mutation m.14459G>A in samples isolated from areas of the aortic intima either affected or unaffected by atherosclerosis. The areas contained different types of atherosclerotic lesions of human aortic intima, namely, fatty streaks and lipofibrous and fibrous plaques, compared to unaffected areas taken from the same autopsy samples. This study revealed that the levels of heteroplasmy of this mutation were significantly higher in the affected areas. Mutation m.G14459A causes a defect in the sixth protein subunit of the mitochondrial respiratory chain enzyme, leading to NADH dehydrogenase dysfunction. The association of this mutation with atherosclerotic lesions is possibly due to the fact that a decrease in the number of normally functioning enzymes in mitochondria leads to oxidative damage in human vascular intima cells. Four other mutations (m.3256C>T, m.12315G>A, m.13513G>A, and m.15059G>A) were shown to be closely associated with the risk of CVD [74]. The described study only revealed associations between the mutational load of mtDNA and presence and severity of atherosclerotic lesions; it did not discuss measurements of mitochondrial function and/or turnover. Furthermore, it remains unclear whether the described mtDNA mutations increase predisposition to atherosclerosis development. Increased mtDNA mutational burden may potentially cause cellular function disruption and formation of oxidative stress conditions at the local level. If mtDNA heteroplasmy is enriched in circulating monocytes/macrophages that are recruited to the lesion site and dwell there [75], the risk of mitochondrial dysfunction and oxidative stress in these sites may be increased. Selective accumulation at the lesion site of cells bearing mtDNA mutations associated with pathology is also possible. Increased mtDNA heteroplasmy of mutations with low minor allele frequencies in the arterial intima affected by atherosclerosis was demonstrated in a recent study [76], which supported the observation that the accumulation of mtDNA mutations is associated with atherosclerotic lesion development, but did not elucidate the mechanistic role of the detected mtDNA mutations or establish causative links. More studies are needed to clarify these questions.

The list of mtDNA mutations associated with cardiovascular diseases in humans is growing. The presence of some of these mutations could be used as predictors of subclinical atherosclerosis and may help disease tracking during early subclinical stages. Moreover, establishing the functional connections between mtDNA mutations and the pathophysiological features of atherosclerosis may help in the development of novel disease therapies.

## 5. Conclusions

Studies regarding mitochondrial genome polymorphisms comprise a rapidly developing research field. Novel associations between mtDNA variants and certain human pathologies are being revealed each year. Cells of the cardiovascular system are characterized by high energy consumption and are strongly dependent on correct mitochondrial function, therefore, it is no surprise that many of the identified mtDNA mutations that disrupt mitochondrial function are associated with cardiovascular diseases. Some of these associations were found to be strong enough to make the diagnostic use of mtDNA mutations possible. Future studies are needed to translate the accumulated knowledge regarding CVD-associated mtDNA mutations into novel therapies for atherosclerosis and related diseases. One direction for such therapies could be the selective targeting of mitochondrial oxidative stress, with correction of mitochondrial dynamics also appearing to be promising. To date, however, mitochondria-targeting therapies for cardiovascular diseases are not being tested clinically and require more preliminary studies to allow this possibility.

## Figures and Tables

**Table 1:** Risk factors of cardiovascular disease and mitochondrial dysfunction.

Risk Factor	Effects on the Mitochondria	Implications in Pathogenesis	References
Vasoactive agents, shear stress	Mitochondrial dysfunction, oxidative stress in VSMCs and ECs, vascular inflammation	Atherosclerosis	[39,40,41]
Vasoactive agents, factors promoting hypertension	Mitochondrial dysfunction in the ECs and cardiomyocytes, impaired ATP production, mitochondrial Ca^2+^ overload	Hypertension aggravation	[39,40]
Hyperlipidemia, hyperglycemia, oxLDL	Mitochondrial dysfunction, oxidative stress and impaired mitophagy in multiple cell types	Diabetes mellitus, atherosclerosis and various CVD	[25,39,42,43,44]
Smoking	Mitochondrial dysfunction, oxidative stress in various vascular cell types, chronic inflammation	Atherosclerosis and various CVD	[45]

CVD, cardiovascular disease; ECs, endothelial cells; oxLDL, oxidized lox-density lipoprotein; VSMCs, vascular smooth muscular cells.

**Table 2:** Mitochondrial mutations associated with cardiovascular disease (CVD) in humans.

Mutation(s)	Location	Effect
m.3243A>G m.3256C>T m.3260A>G	Nucleotide substitutions in the tRNA–Leu gene (UUR recognition codon)	Deficiency of tRNA-Leu, impaired protein synthesis
m.4269A>G m.4300A>G m.4317A>G	Nucleotide substitutions in the tRNA–Ile gene	Deficiency of tRNA-Ile, impaired protein synthesis
m.4833A>G	Subunit 2 of NADH dehydrogenase gene	Defect in protein chain 2 of NADH dehydrogenase, impaired enzyme function
m.8296A>G m.8363G>A	Nucleotide substitutions in the tRNA–Lys gene	Deficiency of tRNA–Lys, impaired protein synthesis
m.1624C>T	Nucleotide substitution in the tRNA–Val gene	Changes in secondary structure and dysfunction of tRNA–Val, decrease associated with hypertrophic cardiomyopathy
m.1541G>A m.1555A>G	Nucleotide substitutions in the 12S rRNA gene	Impaired ribosome function, reduced protein synthesis
m.13513G>A	Subunit 5 of NADH dehydrogenase gene	NADH-dehydrogenase deficiency
m.12192G>A	Nucleotide substitutions in the tRNA–His gene	Deficiency of tRNA–His, impaired protein synthesis shown to be associated with dilated cardiomyopathy
m.9997T>C	Nucleotide substitutions in the tRNA–Gly gene	Deficiency of tRNA-Gly, impaired protein synthesis
m.12297T>C m.12315G>A	Nucleotide substitutions located in the tRNA–Leu gene (recognition codon CUN)	Deficiency of tRNA–Leu. m.12315G>A destroys the highly conserved G–C bases in the TψC stem of the tRNA molecule

## References

[1] Gorman G.S., Chinnery P.F., DiMauro S., Hirano M., Koga Y., McFarland R., Suomalainen A., Thorburn D.R., Zeviani M., Turnbull D.M. (2016). Mitochondrial diseases. Nat. Rev. Dis. Primers.

[2] Kattoor A.J., Pothineni N.V.K., Palagiri D., Mehta J.L. (2017). Oxidative Stress in Atherosclerosis. Curr. Atheroscler. Rep..

[3] Rocha M., Apostolova N., Hernandez-Mijares A., Herance R., Victor V.M. (2010). Oxidative stress and endothelial dysfunction in cardiovascular disease: Mitochondria-targeted therapeutics. Curr. Med. Chem..

[4] Jones D.L., Rodriguez V.J., Alcaide M.L., Barylski N., Cabral D., Rundek T., Weiss S.M., Kumar M. (2017). Subclinical atherosclerosis among young and middle-aged adults using carotid intima-media thickness measurements. South Med. J..

[5] Boovarahan S.R., Kurian G.A. (2018). Mitochondrial dysfunction: A key player in the pathogenesis of cardiovascular diseases linked to air pollution. Rev. Environ. Health.

[6] Sazonova M.A., Sinyov V.V., Ryzhkova A.I., Galitsyna E.V., Khasanova Z.B., Postnov A.Y., Yarygina E.I., Orekhov A.N., Sobenin I.A. (2017). Role of mitochondrial genome mutations in pathogenesis of carotid atherosclerosis. Oxidative Med. Cell. Longev..

[7] Vecoli C., Borghini A., Pulignani S., Mercuri A., Turchi S., Carpeggiani C., Picano E., Andreassi M.G. (2018). Prognostic value of mitochondrial DNA4977 deletion and mitochondrial DNA copy number in patients with stable coronary artery disease. Atherosclerosis.

[8] Heidari M.M., Mirfakhradini F.S., Tayefi F., Ghorbani S., Khatami M., Hadadzadeh M. (2020). Novel point mutations in mitochondrial Mt-Co2 gene may be risk factors for coronary artery disease. Appl. Biochem. Biotechnol..

[9] Rosenson R.S. (2005). HDL-C and the diabetic patient: Target for therapeutic intervention?. Diabetes Res. Clin. Pract..

[10] Org E., Mehrabian M., Lusis A.J. (2015). Unraveling the environmental and genetic interactions in atherosclerosis: Central role of the gut microbiota. Atherosclerosis.

[11] McGillicuddy F.C., Roche H.M. (2012). Nutritional Status, genetic susceptibility, and Insulin Resistance—Important precedents to atherosclerosis. Mol. Nutr. Food Res..

[12] Geovanini G.R., Libby P. (2018). Atherosclerosis and inflammation: Overview and updates. Clin. Sci..

[13] Sobenin I.A., Karagodin V.P., Melnichenko A.C., Bobryshev Y.V., Orekhov A.N. (2013). Diagnostic and prognostic value of low density lipoprotein-containing circulating immune complexes in atherosclerosis. J. Clin. Immunol..

[14] Libby P., Bornfeldt K.E., Tall A.R. (2016). Atherosclerosis: Successes, surprises, and future challenges. Circ. Res..

[15] Madamanchi N.R., Runge M.S. (2007). Mitochondrial Dysfunction in Atherosclerosis. Circ. Res..

[16] Vásquez-Trincado C., García-Carvajal I., Pennanen C., Parra V., Hill J.A., Rothermel B.A., Lavandero S. (2016). Mitochondrial dynamics, mitophagy and cardiovascular disease. J. Physiol..

[17] Navas-Madroñal M., Rodriguez C., Kassan M., Fité J., Escudero J.R., Cañes L., Martínez-González J., Camacho M., Galán M. (2019). Enhanced endoplasmic reticulum and mitochondrial stress in abdominal aortic aneurysm. Clin. Sci. (Lond).

[18] Singh B., Modica-Napolitano J.S., Singh K.K. (2017). Defining the momiome: Promiscuous information transfer by mobile mitochondria and the mitochondrial genome. Semin. Cancer Biol..

[19] Van der Bliek A.M., Sedensky M.M., Morgan P.G. (2017). Cell Biology of the Mitochondrion. Genetics.

[20] Wallace D.C., Chalkia D. (2013). Mitochondrial DNA genetics and the heteroplasmy conundrum in evolution and disease. Cold Spring Harb. Perspect. Biol..

[21] Lee H.C., Wei Y.H. (2005). Mitochondrial biogenesis and mitochondrial DNA maintenance of mammalian cells under oxidative stress. Int. J. Biochem. Cell Biol..

[22] Jeong S.Y., Seol D.W. (2008). The role of mitochondria in apoptosis. BMB Rep..

[23] Sarasija S., Norman K.R. (2015). A γ-Secretase independent role for presenilin in calcium homeostasis impacts mitochondrial function and morphology in caenorhabditis elegans. Genetics.

[24] Kim H.E., Grant A.R., Simic M.S., Kohnz R.A., Nomura D.K., Durieux J., Riera C.E., Sanchez M., Kapernick E., Wolff S. (2016). Lipid biosynthesis coordinates a mitochondrial-to-cytosolic stress response. Cell.

[25] Rocha M., Diaz-Morales N., Rovira-Llopis S., Escribano-Lopez I., Bnuls C., Hernandez-Mijares A., Diamanti-Kandarakis E., Victor V.M. (2016). Mitochondrial dysfunction and endoplasmic reticulum stress in diabetes. Curr. Pharm Des..

[26] Lenaz G., Genova M.L. (2010). Structure and organization of mitochondrial respiratory complexes: A new understanding of an old subject. Antioxid. Redox Signal..

[27] Camara A.K., Lesnefsky E.J., Stowe D.F. (2010). Potential therapeutic benefits of strategies directed to mitochondria. Antioxid Redox Signal..

[28] Szczepanowska K., Trifunovic A. (2017). Origins of mtDNA mutations in ageing. Essays Biochem..

[29] El-Hattab A.W., Craigen W.J., Scaglia F. (2017). Mitochodnrial DNA maintenance defects. Biochim. Biophys. Acta Mol. Basis Dis..

[30] Niemann J., Johne C., Schröder S., Koch F., Ibrahim S.M., Schultz J., Tiedge M., Baltrusch S. (2017). An mtDNA mutation accelerates liver aging by interfering with the ROS response and mitochondrial life cycle. Free Radic. Biol. Med..

[31] Kujoth G.C., Hiona A., Pugh T.D., Someya S., Panzer K., Wohlgemuth S.E., Hofer T., Seo A.Y., Sullivan R., Jobling W.A. (2005). Mitochondrial DNA mutations, oxidative stress, and apoptosis in mammalian aging. Science.

[32] Tilokani L., Nagashima S., Paupe V., Prudent J. (2018). Mitochondrial dynamics: Overview of molecular mechanisms. Essays Biochem..

[33] Bertholet A., Delerue T., Millet A. (2016). Mitochondrial fusion/fission dynamics in neurodegeneration and neuronal plasticity. Neurobiol. Dis..

[34] Waldmeier P.C. (2003). Prospects for antiapoptotic drug therapy of neurodegenerative diseases. Prog. Neuropsychopharmacol. Biol. Psychiatry..

[35] Cooper H.A., Cicalese S., Preston K.J., Kawai T., Okuno K., Choi E.T., Kasahara S., Uchida H.A., Otaka N., Scalia R. (2020). Targeting mitochondrial fission as a potential therapeutic for abdominal aortic aneurysm. Cardiovasc. Res..

[36] Yamada Y., Akita H., Kogure K., Kamiya H., Harashima H. (2007). Mitochondrial drug delivery and mitochondrial disease therapy—An approach to liposome-based delivery targeted to mitochondria. Mitochondrion.

[37] Diaz-Vegas A., Sanchez-Aguilera P., Krycer J.R., Morales P.E., Monsalves-Alvarez M., Cifuentes M., Rothermel B.A., Lavandero S. (2020). Is mitochondrial dysfunction a common root of noncommunicable chronic diseases?. Endocr. Rev..

[38] Siasos G., Tsigkou V., Kosmopoulos M., Theodosiadis D., Simantiris S., Tagkou N.M., Tsimpiktsioglou A., Stampouloglou P.K., Oikonomou E., Mourouzis K. (2018). Mitochondria and cardiovascular diseases-from pathophysiology to treatment. Ann. Transl. Med..

[39] Staiculescu M.C., Foote C., Meininger G.A., Martinez-Lemus L.A. (2014). The role of reactive oxygen species in microvascular remodeling. Int. J. Mol. Sci..

[40] Knight-Lozano C.A., Young C.G., Burow D.L., Hu Z.Y., Uyeminami D., Pinkerton K.E., Ischiropoulos H., Ballinger S.W. (2002). Cigarette smoke exposure and hypercholesterolemia increase mitochondrial damage in cardiovascular tissues. Circulation.

[41] Kovacic J.C., Castellano J.M., Farkouh M.E., Fuster V. (2014). The relationships between cardiovascular disease and diabetes: Focus on pathogenesis. Endocrinol. Metab. Clin. N. Am..

[42] Yao P.M., Tabas I. (2001). Free cholesterol loading of macrophages is associated with widespread mitochondrial dysfunction and activation of the mitochondrial apoptosis pathway. J. Biol. Chem..

[43] Fleming I., Mohamed A., Galle J., Turchanowa L., Brandes R.P., Fisslthaler B., Busse R. (2005). Oxidized low-density lipoprotein increases superoxide production by endothelial nitric oxide synthase by inhibiting PKCalpha. Cardiovasc. Res..

[44] Geng Y.J., Libby P. (2002). Progression of atheroma: A struggle between death and procreation. Arterioscler. Thromb. Vasc. Biol..

[45] Siasos G., Tsigkou V., Kokkou E., Oikonomou E., Vavuranakis M., Vlachopoulos C., Verveniotis A., Limperi M., Genimata V., Papavassiliou A.G. (2014). Smoking and atherosclerosis: Mechanisms of disease and new therapeuticapproaches. Curr. Med. Chem..

[46] Ward N.C., Croft K.D. (2006). Hypertension and oxidative stress. Clin. Exp. Pharmacol. Physiol..

[47] Postnov I.V. (2001). The role of mitochondrial calcium overload and energy deficiency in pathogenesis of arterial hypertension. Arkhiv Patol..

[48] Gioscia-Ryan R.A., LaRocca T.J., Sindler A.L., Zigler M.C., Murphy M.P., Seals D.R. (2014). Mitochondria-targeted antioxidant (MitoQ) ameliorates age-related arterial endothelial dysfunction in mice. J. Physiol..

[49] Vendrov A.E., Stevenson M.D., Alahari S., Pan H., Wickline S.A., Madamanchi N.R., Runge M.S. (2017). Attenuated superoxide dismutase 2 activity induces atherosclerotic plaque instability during aging in hyperlipidemic mice. J. Am. Heart Assoc..

[50] Perrotta I., Brunelli E., Sciangula A., Conforti F., Perrotta E., Tripepi S., Donato G., Cassese M. (2011). iNOS induction and PARP-1 activation in human atherosclerotic lesions: An immunohistochemical and ultrastructural approach. Cardiovasc. Pathol..

[51] Fiorentino T.V., Prioletta A., Zuo P., Folli F. (2013). Hyperglycemia-induced oxidative stress and its role in diabetes mellitus related cardiovascular diseases. Curr. Pharm Des..

[52] Wang P., Wang S.C., Yang H., Lv C., Jia S., Liu X., Wang X., Meng D., Qin D., Zhu H. (2019). Therapeutic Potential of Oxytocin in Atherosclerotic Cardiovascular Disease: Mechanisms and Signaling Pathways. Front Neurosci..

[53] Xiong S., Wang P., Ma L., Gao P., Gong L., Li L., Li Q., Sun F., Zhou X., He H. (2016). Ameliorating endothelial mitochondrial dysfunction restores coronary function via transient receptor potential vanilloid 1-Mediated protein kinase A/Uncoupling Protein 2 Pathway. Hypertension.

[54] Chiaratti M.R., Macabelli C.H., Neto J.D.A., Grejo M.P., Pandy A.K., Perecin F., Del collado M. (2020). Maternal transmission of mitochondrial diseases. Genet. Mol. Biol..

[55] Xu J., Nuno K., Litzenburger U.M., Qi Y., Corces M.R., Majeti R., Chang H.Y. (2019). Single-cell lineage tracing by endogenous mutations enriched in transposase accessible mitochondrial DNA. Elife.

[56] Rossignol R., Faustin B., Rocher C., Malgat M., Mazat J.P., Letellier T. (2003). Mitochondrial threshold effects. Biochem. J..

[57] Sobenin I.A., Sazonova M.A., Postnov A.Y., Bobryshev Y.V., Orekhov A.N. (2013). Changes of mitochondria in atherosclerosis: Possible determinant in the pathogenesis of the disease. Atherosclerosis.

[58] Hefti E., Blanco J.G. (2018). Mitochondrial DNA heteroplasmy in cardiac tissue from individuals with and without coronary artery disease. Mitochondrial DNA A DNA Mapp. Seq. Anal..

[59] Abu-Amero K.K., Osman E.A., Dewedar A.S., Schmidt S., Allingham R.R., Al-Obeidan S.A. (2010). Analysis of LOXL1 polymorphisms in a Saudi Arabian population with pseudoexfoliation glaucoma. Mol. Vis..

[60] Zhang Y., Ma Y., Bu D., Liu H., Xia C., Zhang Y., Zhu S., Pan H., Pei P., Zheng X. (2015). Deletion of a 4977-bp fragment in the mitochondrial genome is associated with mitochondrial disease severity. PLoS ONE.

[61] Weakley S.M., Jiang J., Kougias P., Lin P.H., Yao Q., Brunicardi F.C. (2010). Role of somatic mutations in vascular disease formation. Expert Rev. Mol. Diagn..

[62] Sawabe M., Tanaka M., Chida K., Arai T., Nishigaki Y., Fuku N., Mieno M.N., Kuchiba A., Tanaka N. (2011). Mitochondrial haplogroups A and M7a confer a genetic risk for coronary atherosclerosis in the Japanese elderly: An autopsy study of 1536 patients. J. Atheroscler. Thromb..

[63] Vindis C., Elbaz M., Escargueil-Blanc I., Augé N., Heniquez A., Thiers J.C., Nègre-Salvayre A., Salvayre R. (2005). Two distinct calcium-dependent mitochondrial pathways are involved in oxidized LDL-induced apoptosis. Arterioscler. Thromb. Vasc. Biol..

[64] Liu Y., Gao L., Xue Q., Li Z., Wang L., Chen R., Liu M., Wen Y., Guan M., Li Y. (2011). Voltage-dependent anion channel involved in the mitochondrial calcium cycle of cell lines carrying the mitochondrial DNA A4263G mutation. Biochem. Biophys. Res. Commun..

[65] Chen X., Zhang Y., Xu B., Cai Z., Wang L., Tian J., Liu Y., Li Y. (2016). The mitochondrial calcium uniporter is involved in mitochondrial calcium cycle dysfunction: Underlying mechanism of hypertension associated with mitochondrial tRNA(Ile) A4263G mutation. Int. J. Biochem. Cell Biol..

[66] Zhu Y., Gu X., Xu C. (2018). Mitochondrial DNA 7908-8816 region mutations in maternally inherited essential hypertensive subjects in China. BMC Med. Genom..

[67] Zhu Y., You J., Xu C., Gu X. (2020). Associations of mitochondrial DNA 3777–4679 region mutations with maternally inherited essential hypertensive subjects in China. BMC Med. Genet..

[68] Bornstein B., Mas J.A., Patrono C., Fernández-Moreno M.A., González-Vioque E., Campos Y., Carrozzo R., Martín M.A., del Hoyo P., Santorelli F.M. (2005). Comparative analysis of the pathogenic mechanisms associated with the G8363A and A8296G mutations in the mitochondrial tRNA(Lys) gene. Biochem. J..

[69] Pohjoismäki J.L., Goffart S., Taylor R.W., Turnbull D.M., Suomalainen A., Jacobs H.T., Karhunen P.J. (2010). Developmental and pathological changes in the human cardiac muscle mitochondrial DNA organization, replication and copy number. PLoS ONE.

[70] Chol M., Lebon S., Bénit P., Chretien D., de Lonlay P., Goldenberg A., Odent S., Hertz-Pannier L., Vincent-Delorme C., Cormier-Daire V. (2003). The mitochondrial DNA G13513A MELAS mutation in the NADH dehydrogenase 5 gene is a frequent cause of Leigh-like syndrome with isolated complex I deficiency. J. Med. Genet..

[71] Mimaki M., Ikota A., Sato A., Komaki H., Akanuma J., Nonaka I., Goto Y. (2003). A double mutation (G11778A and G12192A) in mitochondrial DNA associated with Leber’s hereditary optic neuropathy and cardiomyopathy. J. Hum. Genet..

[72] Raha S., Merante F., Shoubridge E., Myint A.T., Tein I., Benson L., Johns T., Robinson B.H. (1999). Repopulation of rho0 cells with mitochondria from a patient with a mitochondrial DNA point mutation in tRNA(Gly) results in respiratory chain dysfunction. Hum. Mutat..

[73] Andreassi M.G., Botto N., Colombo M.G., Biagini A., Clerico A. (2000). Genetic instability and atherosclerosis: Can somatic mutations account for the development of cardiovascular diseases?. Environ. Mol. Mutagenes..

[74] Mitrofanov K.Y., Zhelankin A.V., Shiganova G.M., Sazonova M.A., Bobryshev Y.V., Postnov A.Y., Sobenin I.A., Orekhov A.N. (2016). Analysis of Mitochondrial DNA Heteroplasmic Mutations A1555G, C3256T, T3336C, C5178A;, G12315A, G13513A, G14459A, G14846A and G15059A in CHD Patients With the History of Myocardial Infarction. Exp. Mol. Pathol..

[75] Galkina E., Ley K. (2009). Immune and inflammatory mechanisms of atherosclerosis. Annu. Rev. Immunol..

[76] Sobenin I.A., Zhelankin A.V., Khasanova Z.B., Sinyov V.V., Medvedeva L.V., Sagaidak M.O., Makeev V.J., Kolmychkova K.I., Smirnova A.S., Sukhorukov V.N. (2019). Heteroplasmic variants of mitochondrial DNA in atherosclerotic lesions of human aortic intima. Biomolecules.

